# Active demethylation upregulates CD147 expression promoting non-small cell lung cancer invasion and metastasis

**DOI:** 10.1038/s41388-022-02213-0

**Published:** 2022-02-07

**Authors:** Cheng-Gong Liao, Xiao-Hua Liang, Yuan Ke, Li Yao, Man Liu, Ze-Kun Liu, Lin He, Yi-Xiao Guo, Huijie Bian, Zhi-Nan Chen, Ling-Min Kong

**Affiliations:** 1 Department of Oncology, Tangdu Hospital, Fourth Military Medical University, Xi’an, 710038 People’s Republic of China; 2 Department of Thoracic Surgery, Tangdu Hospital, Fourth Military Medical University, Xi’an, 710038 People’s Republic of China; 3 Department of Pathology, Xi’an No. 3 Hospital, The Affiliated Hospital of Northwest University, Xi’an, 710018 People’s Republic of China; 4 Department of Cell Biology, National Translational Science Center for Molecular Medicine, Fourth Military Medical University, Xi’an, 710032 People’s Republic of China

**Keywords:** Cell migration, Prognostic markers

## Abstract

Non-small cell lung cancer (NSCLC) is a fatal disease, and its metastatic process is poorly understood. Although aberrant methylation is involved in tumor progression, the mechanisms underlying dynamic DNA methylation remain to be elucidated. It is significant to study the molecular mechanism of NSCLC metastasis and identify new biomarkers for NSCLC early diagnosis. Here, we performed MeDIP-seq and hMeDIP-seq analyses to detect the genes regulated by dynamic DNA methylation. Comparison of the 5mC and 5hmC sites revealed that the *CD147* gene underwent active demethylation in NSCLC tissues compared with normal tissues, and this demethylation upregulated CD147 expression. Significantly high levels of CD147 expression and low levels of promoter methylation were observed in NSCLC tissues. Then, we identified the *CD147* promoter as a target of KLF6, MeCP2, and DNMT3A. Treatment of cells with TGF-β triggered active demethylation involving loss of KLF6/MeCP2/DNMT3A and recruitment of Sp1, Tet1, TDG, and SMAD2/3 transcription complexes. A dCas9-SunTag-DNMAT3A-sgCD147-targeted methylation system was constructed to reverse CD147 expression. The targeted methylation system downregulated CD147 expression and inhibited NSCLC proliferation and metastasis in vitro and in vivo. Accordingly, we used cfDNA to detect the levels of *CD147* methylation in NSCLC tissues and found that the *CD147* methylation levels exhibited an inverse relationship with tumor size, lymphatic metastasis, and TNM stage. In conclusion, this study clarified the mechanism of active demethylation of *CD147* and suggested that the targeted methylation of *CD147* could inhibit NSCLC invasion and metastasis, providing a highly promising therapeutic target for NSCLC.

## Introduction

Non-small cell lung cancer (NSCLC) is the most common type of lung cancer and is associated with the highest cancer incidence and mortality rates of cancer worldwide [1]. Distant metastasis, especially bone metastasis, is the main outcome of cancer progression and poor prognosis. Moreover, due to the difficulty in monitoring distant metastases and a lack of effective early diagnosis, patients often miss the optimal treatment window [2, 3]. Therefore, it is essential to explore the molecular mechanisms of NSCLC metastasis and develop new biomarkers for the early diagnosis of NSCLC.

DNA methylation is a crucial epigenetic modification that plays an important role in transcription regulation. Some methylation alterations result in pathological states, particularly cancer development and progression [4]. DNA methylation, which often occurs during the early phases of tumorigenesis, is considered a valid marker for cancer predisposition and can be consistently detected because it tends to occur in some specific regions of the DNA [5]. Increasing lines of evidence show that an aberrant methylation (hypermethylation and hypomethylation) pattern is a consistent epigenetic hallmark of human cancers [6]. Hypomethylation of the promoter is correlated with the overexpression of oncogenes and other genes associated with tumor invasion or metastasis [7]. Moreover, alterations in DNA methylation in tumor cells are also reflected in the circulating cell-free DNA (cfDNA) that is released from tumor tissues into the peripheral blood, which makes cfDNA an ideal noninvasive biomarker for cancer diagnosis and predicting prognosis [8].

CD147, a transmembrane glycoprotein belonging to the immunoglobulin superfamily, is highly expressed in different cancer types, including NSCLC, breast cancer, and hepatocellular carcinoma (HCC) [9, 10]. CD147, which is also called basigin, has four isoforms. Basigin-2 and basigin-3/4 can be detected in multiple normal and tumor tissues, but the average expression level of basigin-2 is obviously higher than those of basigin-3 and basigin-4 [11]. Therefore, most studies related to the *BSG* gene have focused on basigin-2, which is the most predominant splice variant and encodes the well-known adhesion molecule CD147/EMMPRIN. Evolving and compelling evidence shows that CD147 plays a key role in tumor progression and metastasis [12]. Recently, chimeric antigen receptor T-cell immunotherapy targeting CD147 has demonstrated antitumor efficacy for the treatment of patients with HCC [13, 14]. Thus, CD147 has been proposed as a promising target in cancer therapy [15]. We have previously reported that promoter hypomethylation upregulates CD147 expression and is associated with poor prognosis in patients with HCC [16]. However, the demethylation mechanisms of *CD147* in cancer cells remain largely unknown.

DNA demethylation can occur passively depending on DNA replication when the newly synthesized DNA strand remains unmethylated. In addition to the passive loss of DNA methylation during replication, DNA methylation can also be actively reversed by enzymes that act directly on 5-methylcytosine (5mC) from the DNA backbone. This process is known as active DNA demethylation. Active DNA demethylation is catalyzed by enzymes including the ten-eleven translocation (Tet) family members and thymine DNA glycosylase (TDG) [17]. However, whether active demethylation occurs in the promoter of *CD147*, and the enzymes that are involved in this process remain unclear.

To clarify the biological function of DNA-methylation events in human epigenetics, a series of genome editing tools, such as zinc-finger nucleases (ZFNs) [18], transcription activator-like effector (TALE) [19], and CRISPR-Cas9 [6, 20], have been used for targeted epigenome editing by fusion with epigenome-modifying enzymes. Our previous study showed that hypomethylation upregulated CD147 expression and that high expression of CD147 promoted tumor progression [16]. Whether we can reverse CD147 expression by targeted methylation to inhibit tumor progression is worth further exploration. Furthermore, we also found that the level of *CD147* methylation in tumor tissues was associated with tumor progression. We hypothesized that it would be worthwhile to determine whether *CD147* methylation could be detected in cfDNA, which is easier to obtain than that from tumor tissues and could therefore provide a means for evaluating tumor progression more effectively.

In the present study, we identified the genes regulated by dynamic DNA methylation in NSCLC. After analyzing the sequencing data, we demonstrated that the *CD147* gene underwent active demethylation in NSCLC. We further investigated the mechanism of active demethylation in *CD147*, used a targeted methylation system to reverse CD147 expression, and attempted to develop an effective clinical therapeutic target for NSCLC.

## Results

### The *CD147* gene underwent demethylation and therefore showed increased expression in NSCLC compared with normal tissues

**Fig. 1:**
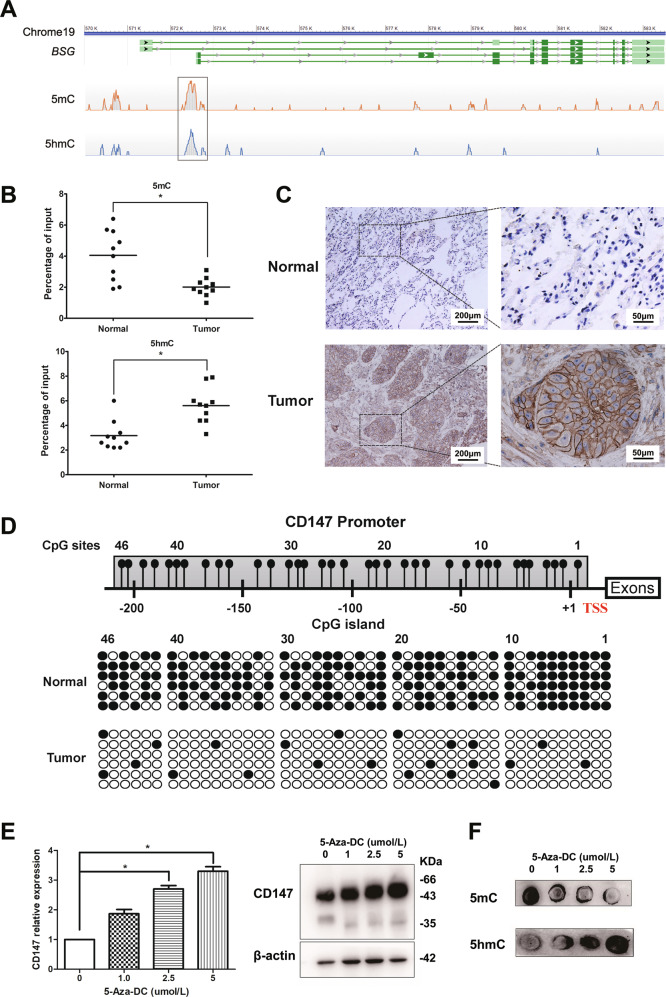
Demethylation upregulated CD147 expression in NSCLC. **A** MeDIP and hMeDIP analysis tracing for 5mC and 5hmC at the *CD147* (BSG) locus. The square indicates that 5mC and 5hmC are located in the same region of the *CD147* promoter. Genes were correlated to fit the UCSC annotations. **B** The content of 5mC and 5hmC in *CD147* promoter were detected using ChIP-qPCR analysis in paired adjacent normal tissues and NSCLC tissues. *P < 0.05, Student’s *t*-test was used for statistical analysis. **C** Representative immunohistochemistry staining of CD147 in paired adjacent normal tissues and NSCLC tissues. **D** Representative methylation profiles of CpG dinucleotides in the *CD147* promoter in adjacent normal tissues and NSCLC tissues detected using bisulfite genomic sequencing. CpG dinucleotides of *CD147* promoter are expressed in roman numerals. Open circles indicate unmethylated CpG dinucleotides and closed circles indicate methylated CpG dinucleotides. The promoter methylation level of *CD147* gene in a sample is expressed as the percentage of methylated CpG sites in all sequenced CpG sites shown in brackets. **E** Demethylation upregulates CD147 expression. CD147 expression was detected using real-time quantitative RT-PCR and western blotting in A549 cells after treatment with different concentrations of 5-Aza-dC. *P < 0.05, One-way ANOVA followed by Dunnett’s test was used for statistical analysis. **F** Genomic 5mC and 5hmC were detected in A549 cells using dot blot analysis after treatment with the indicated concentrations of 5-Aza-dC.

The degree of DNA methylation is reportedly reduced in NSCLC [21]. To define the genes regulated by hypomethylation in NSCLC, we performed ChIP-seq in four paired adjacent normal tissues and NSCLC tissue samples using antibodies directed against 5mC and 5hmC (Supplementary Table 1) [22, 23]. On average, ChIP-seq generated 24137811 raw reads and 23816615 clean reads after filtering out the dirty reads, including low-quality reads, N reads, and adapter sequences. An analysis of the normalized genome-wide distribution of 5mC and 5hmC showed that more than 50% of 5mC and 5hmC located in the intergenic regions, which suggested that methylation regulation mainly occurred in the promoter region between genes (Supplementary Fig. 1 and Supplementary Table 1). When the 5mC and 5hmC sites were overlapped, we found 285 genes that could contain both 5mC and 5hmC (Supplementary Fig. 1). Among these genes, *BSG*, which is also known as *CD147*, is a key gene involved in the development and progression of cancers [12]. Importantly, 5mC/5hmC was detected in the *CD147* promoter region containing Sp1/KLF6 binding sites [24] (Fig. 1A). To further confirm the ChIP-seq data, we examined the 5mC and 5hmC contents in ten pairs of adjacent normal tissues and NSCLC tissue samples. Subsequent qPCR showed that the content of 5mC in the *CD147* promoter in adjacent normal tissues was significantly higher than that in NSCLC tissues, whereas the content of 5hmC exhibited the opposite trend (Fig. 1B). These results indicated that the *CD147* gene underwent increased levels of active demethylation in NSCLC tissues than in normal tissues.

We then explored the relationship between demethylation and gene expression. CD147 protein expression was detected in ten pairs of adjacent normal tissues and NSCLC samples using immunohistochemistry analysis (the information of the NSCLC patients is listed in Supplementary Table 2). The results indicated that CD147 was upregulated in NSCLC tissues, whereas the staining of CD147 in adjacent normal tissues was negative. We also found that CD147 displayed positive membrane and cytoplasm staining, whereas the stroma was not highly stained (Fig. 1C). We found that the expression of CD147 was negatively correlated with the 5mC content in the *CD147* promoter (*r* = −0.6441 and *P* < 0.05), whereas the 5hmC content showed the opposite trend (*r* = 0.6053 and *P* < 0.05, Supplementary Fig. 2). It has been reported that the human *CD147* promoter contains a CpG island close to the transcription start site, which is hypomethylated in HCC [16]. In this study, we assessed the methylation levels of the *CD147* promoter through the bisulfite genomic sequencing (BGS) of lung tissues. Our findings revealed that the methylation levels of NSCLC tissues were markedly lower than those of adjacent normal tissues (12.15 ± 7.14% versus 57.47 ± 12.57%, *P* < 0.05, Fig. 1D). We also detected the CD147 expression and methylation levels in other tumor cells (Supplementary Fig. 3). These results indicated that hypomethylation of the *CD147* promoter region might participate in the increase in CD147 expression observed in NSCLC tissues.

To study the methylation status regulating CD147 expression, the DNA-demethylating reagent 5-Aza-dC was added to A549 cells. The CD147 mRNA and protein expression levels were greatly increased after 5-Aza-dC treatment (Fig. 1E). Additionally, we performed dot blot analysis of bulk genomic DNA using anti-5mC and anti-5hmC antibodies to determine whether 5-Aza-dC could induce global changes in DNA demethylation. Although this experiment was only semiquantitative, we consistently observed obvious decreases in DNA methylation after 5-Aza-dC treatment (Fig. 1F). Remarkably, the 5hmC content was increased after treatment, which suggested that the effects of 5-Aza-dC on DNA demethylation were achieved through active demethylation. The above results suggest that the *CD147* gene had underwent active demethylation changes in NSCLC, and the observed decrease in methylation upregulated CD147 expression.

### The transcriptional regulatory complex of the *CD147* promoter differed between NSCLC and normal tissues

**Fig. 2:**
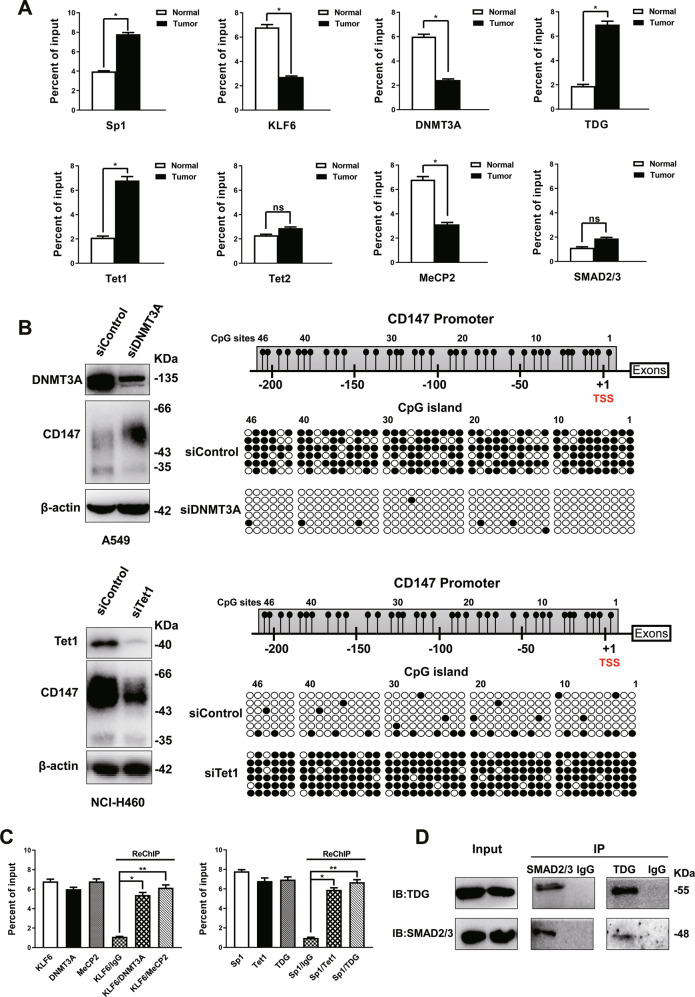
The transcriptional regulatory complex of *CD147* gene changed from normal to NSCLC tissues. **A** The *CD147* promoter was analyzed by ChIP-qPCR using the indicated antibodies. *P < 0.05, **P < 0.01, Student’s *t*-test was used for s*t*atistical analysis. **B** Knockdown of DNMT3A or Tet1 using siRNA. Western blotting was performed to detect protein expression with the indicated siRNA in A549 and NCI-H460 cells. Right panel: *CD147* promoter methylation status after knockdown using bisulfite genomic sequencing. **C** ChIP or sequential ChIP (ChIP-ReChIP) was performed using the indicated antibodies. *P < 0.05, one-way ANOVA followed by Dunnett’s test was used for statistical analysis. **D** Interaction of SMAD2/3-TDG in NCI-H460 cells determined using co-IP assay.

The CpG island of the *CD147* promoter also contains the Sp1/KLF6 binding region [24]. Therefore, we investigated the correlation between DNA methylation regulation and transcription factor regulation in the *CD147* promoter. Using ChIP-qPCR, we examined Sp1/KLF6 binding in normal and NSCLC tissues. The recruitment of Sp1 was dramatically increased in NSCLC compared with normal tissues, whereas KLF6 binding was substantially reduced (Fig. 2A), suggesting that the transcription factor had changed between normal and lung cancer tissues. These results were consistent with our earlier observations [12, 24] that both Sp1 and KLF6 could bind to the *CD147* promoter. The binding of the *de novo* methyltransferase DNMT3A which is usually associated with repression at specific genomic loci, was also detected [25]. The binding of DNMT3A in normal tissues was considerably higher than that in NSCLC (Fig. 2A). Thus, *CD147* was hypermethylated in normal tissues and its expression was low. The ChIP-seq results indicated that the 5hmC and 5mC levels of *CD147* were increased and decreased, respectively, in NSCLC, which indicated that DNA demethylation was an active demethylation process. We then attempted to reveal whether the key demethylation enzymes Tet1 and Tet2 were involved in the regulation of the active demethylation of *CD147*. Our results indicated that Tet1 rather than Tet2 could specifically bind to the *CD147* promoter and that the binding of Tet1 was increased in NSCLC (Fig. 2A), which suggested that Tet1 might regulate the active demethylation process of *CD147*.

Subsequently, to assess the influence of DNMT3A and Tet1 on *CD147* gene methylation, we performed knockdown experiments and detected the *CD147* promoter methylation status by BGS. The hypermethylation of the *CD147* promoter was reversed by knockdown of *DNMT3A* using siRNA, and CD147 expression was increased in A549 cells that expressed relatively low levels of CD147 (Fig. 2B and Supplementary Fig. 4). The knockdown of *Tet1* in NCI-H460 cells that expressed a high level of CD147 (Supplementary Fig. 4) abrogated promoter demethylation, which coincided with decreased CD147 expression (Fig. 2B). We performed knockdown of *Tet1* in A549 and knockdown of *DNMT3A* in NCL-H460 and determined CD147 expression (Supplementary Fig. 5). These observations suggested that DNMT3A was necessary for maintaining the methylation level of *CD147* and that Tet1 mediated active demethylation in the tumor cells.

The combination of other elements, such as MeCP2, which is a member of the methyl-CpG binding domain (MBD) protein family, was tested. MeCP2 could specifically bind to the *CD147* promoter, and the binding was reduced in NSCLC compared with normal lung tissues; this trend was consistent with the results obtained for KLF6 and DNMT3A (Fig. 2A). ChIP-reChIP was performed to confirm whether KLF6, DNMT3A, and MeCP2 could simultaneously bind to the *CD147* promoter. First, chromatin was immunoprecipitated with an anti-DNMT3A or anti-MeCP2 antibody. An additional immunoprecipitation step with an anti-KLF6 specific antibody was then performed using the immunoprecipitated material. Our findings suggested that on the *CD147* promoter, KLF6 combined concurrently with both DNMT3A and MeCP2 to form a complex to regulate CD147 expression in normal tissues (Fig. 2C).

We also tested the recruitment of the TDG protein because recent studies have shown that this protein physically interacts with the Tet family proteins and that Tet1 and TDG jointly occupy similar genomic regions in ESCs [26, 27]. As shown in Fig. 2A, TDG recruitment was markedly increased in NSCLC. Using ChIP-reChIP, we confirmed that Sp1combined with TDG and Tet1 on the same region of the *CD147* promoter (Fig. 2C). These data suggested that the binding of the Sp1/Tet1/TDG complex resulted in a hypomethylated state and increased CD147 expression.

It has been reported that a multiprotein complex involving SMAD2/3 and TDG can regulate gene expression [28]. Our results indicated that SMAD2/3 could not bind to the *CD147* promoter in either normal or NSCLC tissues (Fig. 2A). The co-IP assay showed that SMAD2/3-TDG interaction in NCI-H460 cells (Fig. 2D). In our previous studies, we showed that SMAD regulates CD147 expression [29, 30]. These results strongly suggested that SMAD2/3 interacted with TDG and participated in the formation of the regulatory complexes of *CD147*.

Collectively, these data suggested that the regulatory complex regulating CD147 expression in normal and NSCLC tissues could change and thereby alter the methylation level of *CD147* to regulate gene expression levels.

### TGF-β stimulated the active demethylation of *CD147* by recruiting Tet1 and TDG

**Fig. 3:**
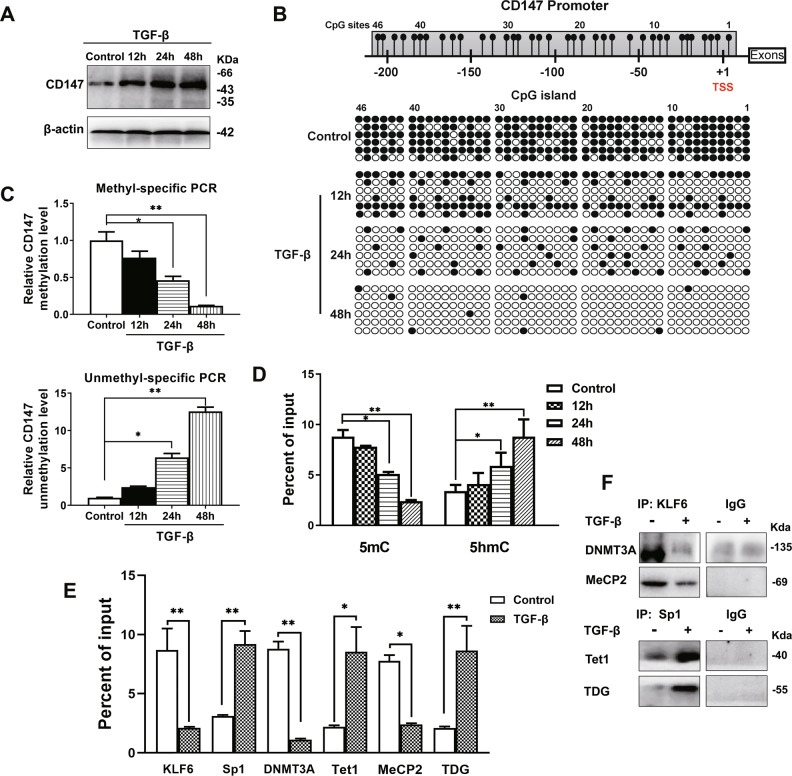
TGF-β induced active demethylation of the *CD147* promoter. **A** The CD147 expression was detected after treatment with 20 ng/mL TGF-β for various time periods. **B** *CD147* promoter methylation status was examined using bisulfite genomic fsequencing. **C** Methyl-specific and unmethyl-specific PCR were performed using treated samples as described in (**A**). **D** ChIP-qPCR was used to detect the 5mC and 5hmC content of *CD147* promoter after TGF-β treatment. The above experiments were carried out in A549 cells. *P < 0.05, **P < 0.01, one-way ANOVA followed by Dunnett’s test was used for statistical analysis in (**C**) and (**D**). **E** TGF-β induced the regulatory complex switch at the *CD147* promoter. ChIP-qPCR was performed with the indicated antibodies after TGF-β treatment of A549 cells for 24 h. *P < 0.05, **P < 0.01, Student’s *t*-test was used for statistical analysis. **F** Interactions of KLF6-DNMT3A, KLF6-MeCP2, Sp1-Tet1, and Sp1-TDG in A549 cells treated with TGF-β were determined using co-IP assay.

We found that SMAD2/3, which is a molecular involved in the TGF-β pathway, participated in the formation of the regulatory complex of *CD147*. However, whether TGF-β induces changes in the regulatory complexes should be further clarified. Accordingly, A549 cells were treated with TGF-β for several durations and collected for western blotting, BGS, and methyl-specific and unmethyl-specific PCR assays. First, CD147 protein expression increased with increase in the TGF-β treatment duration (Fig. 3A). Moreover, demethylation was detected in the core CpG island, whereas almost complete demethylation was observed by 48 h (Fig. 3B, C). Furthermore, we found that this demethylation was completed in the form of active demethylation. ChIP-qPCR results showed that the 5hmC and 5mC levels increased and decreased after TGF-β treatment, respectively (Fig. 3D). Subsequently, ChIP-qPCR of the *CD147* promoter of A549 cells treated with TGF-β were performed, and the results indicated that KLF6, DNMT3A, and MeCP2 binding were reduced, whereas the binding of Sp1, Tet1, and TDG to the same promoter region were increased (Fig. 3E). Co-IP assays showed that TGF-β treatment decreased the binding of KLF6 to DNMT3A and MeCP2 and increased the binding of Sp1 to TDG and Tet1 (Fig. 3F). These findings suggested that TGF-β initiated active demethylation by altering the *CD147* transcription complex to upregulate CD147 expression.

**Fig. 4:**
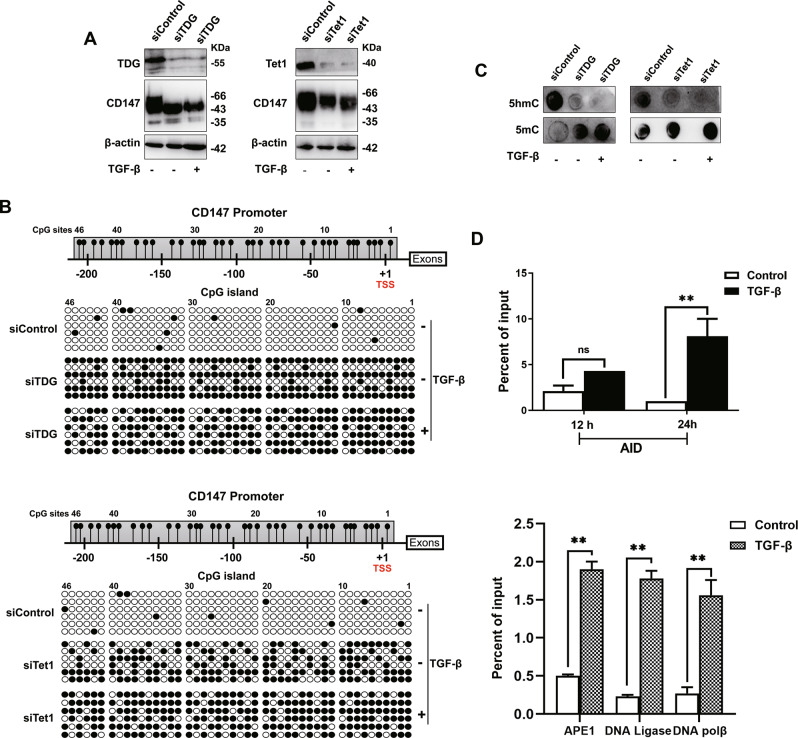
TGF-β stimulated active demethylation of *CD147* by recruiting Tet1 and TDG. **A** and **B** Protein expression of Tet1, TDG, and CD147 was detected using western blotting after transfecting with *Tet1* and *TDG* siRNA and the addition of TGF-β. Subsequently, the *CD147* promoter methylation status was analyzed using bisulfite genomic sequencing. **C** Genomic 5mC and 5hmC levels were determined using dot blot analysis after treatment as described in (**A**). **D** AID and components of the BER machinery were recruited to the *CD147* promoter detected by ChIP-qPCR using the indicated antibodies. The above experiments were carried out in NCI-H460 cells. **P < 0.01, Student’s *t*-test was used for statistical analysis.

To assess whether Tet1 and TDG are essential for TGF-β-dependent demethylation of the *CD147* promoter, *Tet1-* or *TDG*-specific siRNA was transfected into NCI-H460 cells before TGF-β treatment. Tet1 and TDG knockdown markedly inhibited the demethylation of the *CD147* promoter and decreased CD147 protein expression, and this reduction could not be reversed by the addition of TGF-β (Fig. 4A, B). Furthermore, we performed the dot blot analysis using anti-5mC and anti-5hmC antibodies after Tet1 and TDG knockdown and found that Tet1 and TDG knockdown increased the 5mC levels and reduced 5hmC levels and that demethylation could not be reversed following TGF-β treatment (Fig. 4C). This finding indicated that the effect of TGF-β on DNA demethylation was on the whole genome and dependent on Tet1 and TDG.

We then demonstrated that Activation-induced deaminase (AID) recruited to the *CD147* promoter region responded to TGF-β. Additionally, the base-excision repair (BER) enzymes, DNA polymerase (pol) β, apyrimidinic endonuclease-I (APE-1), and DNA ligase I were recruited to the same region after TGF-β treatment, which was consistent with the demethylation requirement of the active promoter in the BER pathway (Fig. 4D). Through the action of these enzymes, the active demethylation of the *CD147* promoter was completed.

Altogether, these results supported the notion that TGF-β-induced active demethylation recruiting Sp1, Tet1, TDG, and the SMAD2/3 transcription complex, leads to CD147 overexpression in NSCLC compared with normal lung tissues.

### Targeted methylation of *CD147* in NSCLC cells downregulated CD147 expression with dCas9-SunTag-DNMAT3A-sgCD147 system

**Fig. 5:**
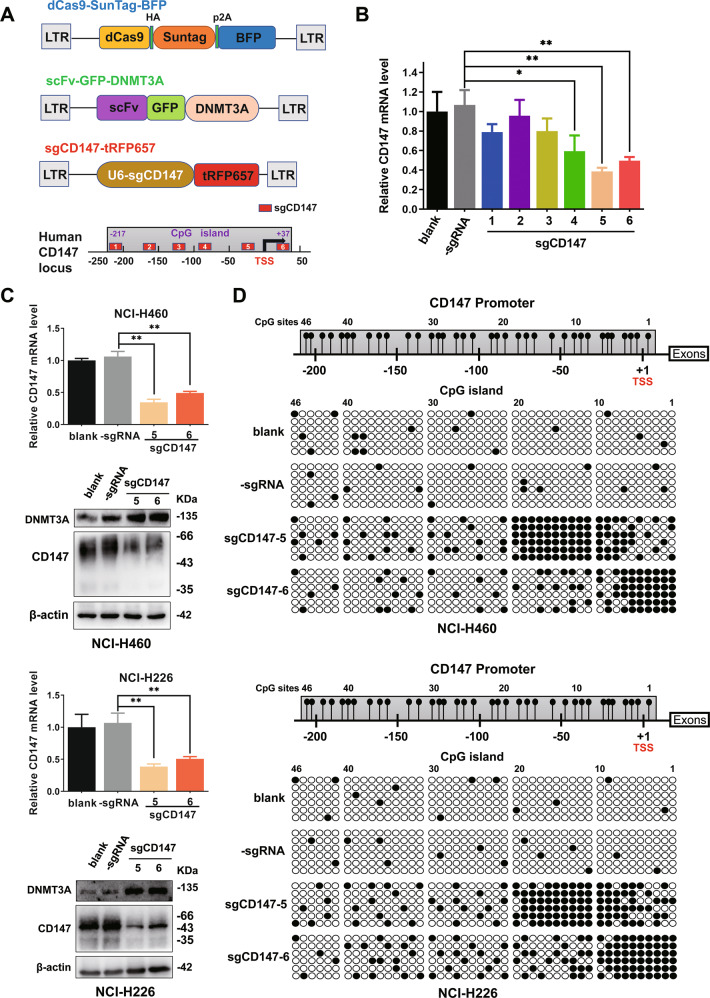
Targeted methylation of *CD147* in NSCLC cells downregulated CD147 expression with dCas9-SunTag-DNMAT3A-sgCD147 system. **A** Schematic description of targeted methylation via dCas9-SunTag-DNMAT3A-sgCD147. The sgRNAs recognizing respective target sites are shown in red. **B** *CD147* expression was detected using quantitative real-time PCR after dCas9-SunTag-DNMAT3A-sgCD147 (1–6) transfection. **C** CD147 expression levels were detected using quantitative real-time PCR and western blotting after transfection using the dCas9-SunTag-DNMAT3A-sgCD147 system. DNMT3A expression was also detected using western blotting. *P < 0.05, **P < 0.01, one-way ANOVA followed by Dunnett’s test was used for statistical analysis in (**A**) and (**B**). **D** Subsequently, *CD147* promoter methylation status was analyzed using bisulfite genomic sequencing.

To reverse CD147 expression by targeting methylation of CD147, we constructed a dCas9-SunTag-DNMAT3A-sgCD147 specific methylation system [6]. After transfection of NCI-H460 cells with the dCas9-SunTag-DNMAT3A-sgCD147 system, the highest decrease in *CD147* transcription was detected with sgCD147-4, sgCD147-5, and sgCD147-6 (Fig. 5A, B). Among these, the dCas9-SunTag-DNMAT3A-sgCD147-5 and dCas9-SunTag-DNMAT3A-sgCD147-6 systems, which exhibited the highest decrease, were chosen for further studies. The dCas9-SunTag-DNMAT3A-sgCD147-5 and dCas9-SunTag-DNMAT3A-sgCD147-6 systems were then transfected into NCI-H460 and NCI-H226 cells, respectively, and quantitative real-time PCR and western blotting revealed that CD147 expression was significantly decreased (Fig. 5C). BGS also demonstrated that more CpG sites in the *CD147* promoter were methylated (Fig. 5D). Therefore, we successfully constructed a methylation system targeting *CD147* and reduced the expression of CD147 in NSCLC cells.

### Targeted methylation of *CD147* inhibited the invasive, metastatic, and proliferative capacities of NSCLC cells in vitro

**Fig. 6:**
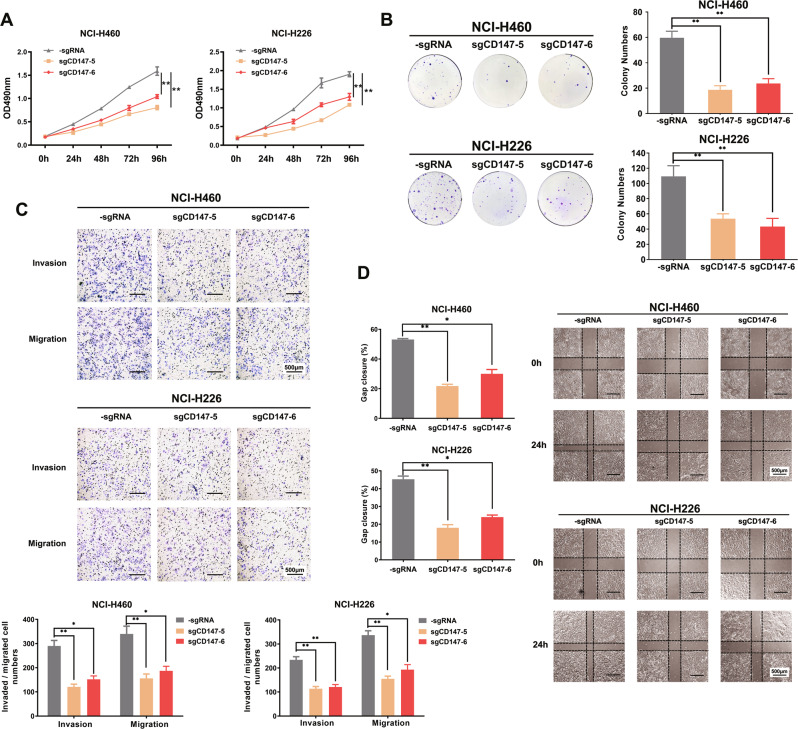
Targeted methylation of *CD147* inhibited NSCLC progression in vitro. **A** MTT assay was performed to detect the proliferation of NSCLC cells transfected with targeted methylation vector at the indicated time periods. *P < 0.05, **P < 0.01. Two-way repeated-measures ANOVA followed by Bonferroni test was used for statistical analysis. **B** Colony-formation assay was performed using cells after transfection as described. Colony numbers per well were counted. **C** Effects of targeted methylation of *CD147* on the invasion and migration of NSCLC cells were detected using Transwell assay. **D** A wound-healing assay was performed using transfected NSCLC cells. *P < 0.05, **P < 0.01, one-way ANOVA followed by Dunnett’s test was used for statistical analysis in (**B**), (**C**) and (**D**).

We then detected the effect of targeted methylation of *CD147* on the behaviors of lung cancer cells in vitro. Using MTT, we found that the targeted methylation of *CD147* inhibited the proliferation of NSCLC cells (Fig. 6A). As shown in Fig. 6B, lung cancer cells with *CD147*-targeted methylation system formed fewer colonies. Moreover, the migration and invasion of NCI-H460 and NCI-H226 cells transfected with the dCas9-SunTag-DNMAT3A-sgCD147 system were significantly lower than those of the control cells (Fig. 6C). In addition, the results of the wound-healing assay revealed that the targeted methylation of *CD147* could considerably inhibit NSCLC cell motility compared with that obtained with the control groups (Fig. 6D). These findings indicated that the targeted methylation of *CD147* exerts an antitumor effect in NSCLC.

### Targeted methylation of *CD147* inhibited NSCLC proliferation and bone metastasis in vivo

**Fig. 7:**
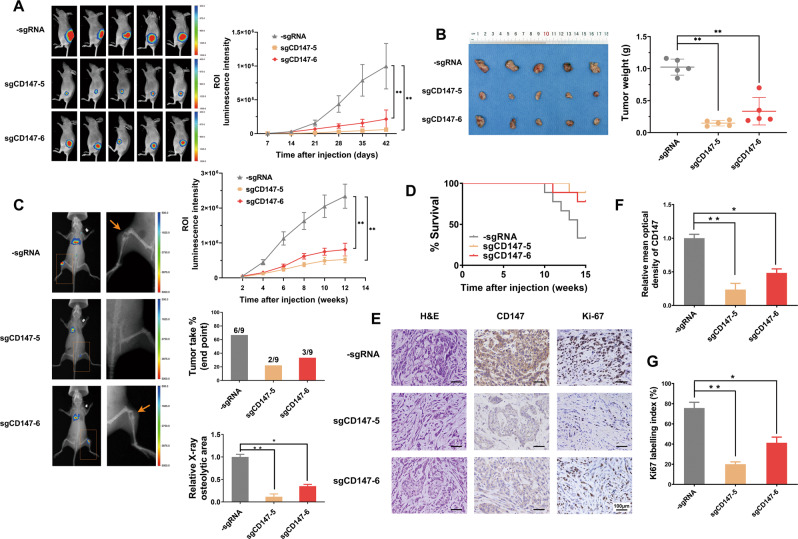
Targeted methylation of *CD147* inhibited NSCLC proliferation and bone metastasis in vivo. **A** Bioluminescence image (BLI) of lung cancer model in orthotopic nude mice model. Left: representative BLI showing the tumor size with the indicated NCI-H460 cells in the mouse model. Right: quantitative analysis of the luminescence intensities and tumor volumes. *n* = 5 mice per group. **P < 0.01, two-way repeated-measures ANOVA followed by Bonferroni test and one-way ANOVA followed by Dunnett’s test were used for statistical analysis. **B** Quantification of the weight of the generated tumors. **P < 0.01, one-way ANOVA followed by Dunnett’s test was used for statistical analysis. **C** Bone metastasis was detected after implantation of NCI-H460 cells in nude mice. *n* = 9 mice per group. Left: BLI and X-ray images. Right: Luminescence intensities were measured every two weeks after implantation. **P < 0.01, two-way repeated-measures ANOVA followed by Bonferroni test were used for statistical analysis. X-ray analysis was used to evaluate tumor incidence and quantify the osteolytic regions in the hindlimbs. Inset numbers represent the number of mice with osteolytic lesions/airway-implanted mice. *P < 0.05, **P < 0.001, one-way ANOVA followed by Dunnett’s test was used for statistical analysis. **D** Survival analysis of mice injected with NCI-H460 sgRNA control and targeted methylation of *CD147* cells (-sgRNA vs sgCD147-5, *P* = 0.0177; -sgRNA *vs* sgCD147-6, *P* = 0.0616). **E** Representative H&E staining of tumor tissues excised from nude mice. CD147 and Ki67 expression was detected using immunohistochemistry. **F** CD147 staining intensity in three groups. The optical density relative to the control group was used to calculate CD147 expression. **G** The percentage of positively stained nuclei was used to calculate Ki67 expression. *P < 0.05; **P < 0.001, one-way ANOVA followed by Dunnett’s test was used for statistical analysis in (**F**) and (**G**).

The in vivo function of targeted methylation of *CD147* in lung cancer was then tested using the xenograft mouse model. NCI-H460 cells with or without the targeted methylation vector were injected into the fat pads in the back of nude mice. We found that targeted methylation of *CD147* had a marked impact on the growth of the primary tumor (Fig. 7A, B), which suggested that targeted methylation of *CD147* exerted a significant inhibitory effect on tumor growth in vivo.

The effect of targeted methylation of *CD147* on the growth of metastatic tumors in distant organs was subsequently examined. NSCLC cells were injected into the airways of nude mice airway to form orthotopic lung cancer. Bone metastasis is one of the most common secondary lesions of lung cancer [3]. In the control mice, NCI-H460 cells caused severe osteolytic bone lesions and massive bone destruction in the femurs. However, cells showing the targeted methylation of *CD147* exhibited less bone damage and tumor lesions (Fig. 7C). Cancer cells with targeted methylation of *CD147* significantly reduced the metastasis signals in limbs. Additionally, the groups with targeted methylation of *CD147* presented less bone metastasis and a reduced osteolytic area compared with the control group (Fig. 7C). Moreover, the targeted methylation of *CD147* significantly prolonged the survival time of mice after cancer cell implantation. Six of 9 (66.7%) mice died of a severe metastasis burden 15 weeks after the implantation of control NCI-H460 cells, whereas only 1 of 9 (11.1%) and 2 of 9 (22.2%) mice in the groups with targeted methylation of *CD147* had died at the same time point, respectively (Fig. 7D). The tumor tissues were excised, and all the samples were examined by H&E staining. IHC staining revealed that the expression of CD147 in the control groups was higher than that in the groups with targeted methylation of *CD147* (Fig. 7E, F). Additionally, Ki67 staining revealed that the targeted methylation of *CD147* led to decreased tumor proliferation (Fig. 7E, G). Together, these findings suggested that the targeted methylation of *CD147* strongly inhibited the proliferation and metastasis of NSCLC in vivo.

### Correlations among *CD147* methylation, tumor grade, and metastatic status of patients with NSCLC

To explore the role of *CD147* methylation in the progression of NSCLC, the *CD147* methylation levels were detected using cfDNA from patients with NSCLC, and the relationships between *CD147* methylation levels and the clinical characteristics of patients were determined. No correlation was found between *CD147* methylation levels and sex, age, or smoking status (*P* > 0.05), and *CD147* methylation levels were inversely associated with tumor size, lymphatic metastasis, and TNM stage (*P* < 0.05) (Supplementary Table 3). Then, we detected the CD147 expression in the primary lung cancer tissues by IHC and found CD147-positive expression in 73.9% of NSCLC tissues (34 of 46, Supplementary Fig. 6A). Combined with the *CD147* methylation levels identified from patients’ cfDNA, Spearman’s correlation analysis revealed a negative relationship between the *CD147* methylation levels and the CD147 protein level (*r* = −0.6809 and *P* < 0.0001; Supplementary Fig. 6B). The above results suggested that *CD147* methylation levels were inversely associated with the degree of NSCLC malignancy and that the status of methylation could be used to predict NSCLC progression.

## Discussion

**Fig. 8:**
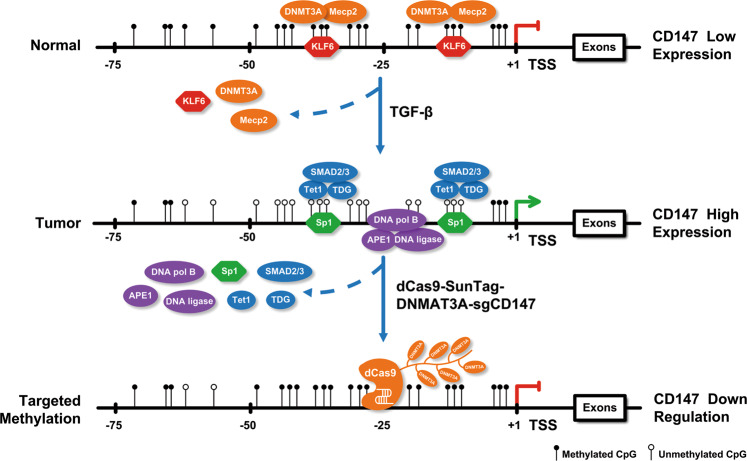
Schematic representation of the mechanism of TGF-β-induced active demethylation of the *CD147* promoter. KLF6/MeCP2/DNMT3A complex bound to the *CD147* promoter resulted in promoter hypermethylation in normal lung cells. TGF-β stimulation resulted in the release of the KLF6/MeCP2/DNMT3A complex and the binding of activating transcription factors involving Sp1, TDG, Tet1, and SMAD2/3. Then, 5mC was oxidized by a series of enzymes to form 5fmC, which was removed by the BER enzymes, and unmethylated cytosine was reintroduced. A dCas9-SunTag-DNMAT3A-sgCD147 specific methylation system was constructed. Using guide RNAs, multiple copies of DNMT3A were directed to specific loci to methylate regions of the *CD147* promoter, which alters the transcription complex to reduce CD147 expression.

In this study, we performed ChIP-seq in four paired adjacent normal tissues and NSCLC tissue samples using antibodies directed against 5mC and 5hmC. A comparison of the 5mC and 5hmC sites led to the identification of the *CD147* gene involved in tumorigenesis. We then confirmed that compared with normal tissues, the *CD147* gene in NSCLC tissues underwent an active demethylation process, and the decreased methylation led to the upregulation of CD147 expression. Furthermore, we found that the transcriptional regulatory complex of the *CD147* gene differed between NSCLC tissues and normal tissues. In normal tissues, KLF6 combined with DNMT3A and MeCP2 to form a complex to regulate CD147 expression on the *CD147* promoter. However, the combination of Sp1/Tet1/TDG/SMAD2/3 complex resulted in a hypomethylated state, which led to an increase in CD147 expression in NSCLC tissues. The conversion of regulatory complexes could be induced by TGF-β, which initiated active demethylation to upregulate CD147 expression. Furthermore, we constructed a dCas9-SunTag-DNMAT3A-sgCD147 specific methylation system to reverse CD147 expression. Our results showed that the targeted methylation of *CD147* downregulated CD147 expression and inhibited NSCLC proliferation and metastasis both in vitro and in vivo. The regulatory network diagram is shown in Fig. 8. Additionally, the cfDNA of patients with NSCLC was collected to determine the relationship between *CD147* methylation levels and the clinical characteristics. We found that the *CD147* methylation levels were inversely related to lymphatic metastasis, tumor size, and TNM stage. Our study clarified the mechanism of active demethylation of *CD147* and suggested that the targeted methylation of *CD147* could inhibit NSCLC invasion and metastasis.

The expression of many tumor-related genes is regulated by dynamic DNA methylation, which regulates tumor progression. Hence, DNA methylation could be a potential epigenetic biomarker for many types of cancer [31]. Here, we attempted to define the genes regulated by DNA methylation in NSCLC. Combined with our ChIP-seq results and our previous studies, we identified that the *CD147* gene regulated by DNA methylation might play an important role in NSCLC progression. We found that *CD147* contained both 5mC and 5hmC, and that the content of 5mC in the *CD147* promoter was markedly higher in adjacent normal tissue than in NSCLC tissues, whereas the opposite trend was observed for 5hmC. Because 5hmC is an intermediary product of active demethylation [32], it can be deaminated to 5-hydroxmethyluracil (5hmU), 5-formylcytosine (5fC) or 5-carboxylcytosine (5cC), which creates a mismatch that is efficiently recognized and excised by TDG, and ultimately regenerates unmodified C [33–36]. Our results showed that the content of 5hmC in the *CD147* promoter was significantly higher in NSCLC tissues than in adjacent normal tissues. These results indicated that the *CD147* gene underwent active demethylation in NSCLC tissues compared with adjacent normal tissues. Moreover, we confirmed that the increased CD147 expression found in NSCLC tissues was induced by hypomethylation of the *CD147* promoter region. We previously demonstrated that the *CD147* promoter is hypomethylated in tumors, and this study reveals that the *CD147* promoter undergoes active demethylation in NSCLC.

Subsequently, a mechanism involving the absence of the KLF6/DNMT3A/MeCP2 complex and recruitment of Sp1, Tet1, TDG, and SMAD2/3 binding to the *CD147* promoter region was identified. Conversion of the regulatory complexes could be induced by TGF-β, which initiated active demethylation to upregulate CD147 expression. The gene that encodes the essential cytokine TGF-β changes from a tumor suppressor gene to a tumor promoter gene during the early stages of carcinogenesis [37, 38]. TGF-β induced lung cancer progression is associated with epigenetic regulation [39–41]. We have previously shown that TGF-β can upregulate CD147 expression to induce epithelial–mesenchymal transition in HCC [42]. In this study, we showed that TGF-β triggered active demethylation and recruited the related BER enzymes to the *CD147* promoter. Furthermore, we found that the active demethylation enzymes Tet1 and TDG bind to the same region of the *CD147* promoter. Importantly, *Tet1* and *TDG* knockdown prevented TGF-β-dependent active demethylation, which indicated that the effects of TGF-β on DNA demethylation depended on Tet1 and TDG. Our work reveals a new pathway through which TGF-β induces active demethylation to upregulate CD147 expression and thus promote NSCLC progression, which could help us better understand the TGF-β signaling pathways. Of course, the regulation of CD147 expression also involves microRNA regulation [12] and posttranscriptional modification [43], but our results show that the DNA demethylation-mediated regulation of the binding of Sp1/Tet1/TDG/SMAD2/3 complex plays an important role in regulating CD147 expression. Our results highlight the dynamic nature of DNA methylation/demethylation and suggest that coregulator balance is the key determinant of *CD147* promoter methylation status.

Although DNA methylation is considered to play an important role in various processes, its causal effects are unclear in many instances due to the lack of widely used techniques to increase and eliminate DNA methylation at specific loci [44]. Therefore, it is necessary and urgent to develop targeted methylation strategies. With the development of epigenome editing tools, ZFNs, TALEs, or the CRISPR-Cas9 system can be used as targeting domains [45]. In the ZFN and TALEN systems, each locus needs the design of endonucleases and protein engineering, which are inefficient [46]. Alternatively, CRISPR RNA-guided Cas9 nuclease target sites use small base-pairing guide RNAs (gRNAs) and then split DNA fragments in a sequence-specific mode. In this study, we established a dCas9-SunTag-DNMAT3A-sgCD147 specific methylation system to recruit DNMT3A to the promoter region and thus remodel DNA methylation [20]. Our results demonstrated that endogenous CD147 expression was efficiently decreased by the dCas9-SunTag-DNMAT3A-sgCD147-5 and dCas9-SunTag-DNMAT3A-sgCD147-6 systems. Furthermore, NSCLC proliferation and metastasis were significantly inhibited by the targeted methylation of *CD147* in vitro and in vivo. Therefore, the dCas9-SunTag-DNMAT3A-sgCD147 system was found to be an effective and precise tool to decrease CD147 expression and inhibit the progression of NSCLC.

cfDNA is a DNA fragment that originates from cell death in tissues and is found in circulating blood [47, 48]. A higher release of cfDNA into the circulation is associated with a greater potential of metastasis, which thereby enhances the ability to distinguish the biotic and clinical variations of the origin of the tumor [49]. Because of the chemical stability of DNA methylation, the fragmented cfDNA can store DNA methylation locations in the gDNA and can be used for the potential detection by noninvasive sampling [50]. Accordingly, we used cfDNA to detect the levels of *CD147* methylation in NSCLC patients and found that the *CD147* methylation levels exhibited an inverse relationship with tumor size, lymphatic metastasis, and TNM stage. Currently, the emergence of new cfDNA biomarkers holds great promise for clinical diagnosis and prognosis determination [51]. Our results might provide a possibility to develop more convenient clinical biomarkers.

To summarize, our study clarified the mechanism of active demethylation of *CD147* in NSCLC and revealed that the Sp1/Tet1/TDG/SMAD2/3 complex played an important role in TGF-β-triggered active demethylation. Targeting DNA methylation can reduce CD147 expression to overcome the aggressive behaviors of proliferation, migration, and invasion in NSCLC. Our findings provide an important insight into the molecular mechanism through which active demethylation upregulates CD147 expression to promote the progression of NSCLC, and may, therefore, contribute to the development of new therapeutic targets in the management of cancer.

## Material and methods

### Collection of tissue specimens

NSCLC and adjacent normal tissues were obtained from surgical specimens from the Department of Thoracic Surgery and the Department of Oncology, Tangdu Hospital of Fourth Military Medical University. This study was approved by the Hospital Ethics Committee and all individuals provided written informed consent. The samples were frozen in liquid nitrogen immediately after surgical removal and maintained at –80 °C until use.

A total of 46 NSCLC specimens were collected from the Department of Oncology, Tangdu Hospital. Circulating cfDNA was prepared using peripheral blood collected from patients following a published procedure [49].

### Methylated and hydroxymethylated DNA immunoprecipitation sequencing (MeDIP and hMeDIP analysis)

Modified chromatin immunoprecipitation (ChIP) assays were performed as previously described [28] using antibodies against 5mC (61255), 5-hydroxymethylcytosine (5hmC, 39769) (Active Motif, La Hulpe, Belgium), or control IgG (sc-2025, Santa Cruz, CA, USA). In-depth whole-genome DNA sequencing was performed by BGI (Shenzhen, China). The obtained reads were then aligned to the *Hg19* human reference genome using Bowtie 2. Peaks were identified using CisGenome and MACS [28]. Enriched binding peaks were generated after filtering through the control input.

### Immunohistochemical (IHC) analysis

The specimens were incubated with a monoclonal antibody against human CD147 (prepared by our lab) [11, 52, 53]. Immunostaining was performed using a Histostain-SP kit (Invitrogen, Carlsbad, CA, USA) according to the manufacturer’s instructions. Immunopositivity was independently evaluated by two pathologists who were blinded to the clinical data. The immunopositivity was evaluated as described in the Supplementary Materials.

### Bisulfite genomic sequencing (BGS)

Using QIAamp DNA mini kit (Qiagen, Valencia, CA, USA), the whole genome DNA was extracted from tissues. Bisulfite conversion was then performed using an EpiTect Bisulfite kit (Qiagen). The CpG island region in the *CD147* promoter was amplified with the bisulfite-treated DNA as the template using an Advantage GC Genomic PCR kit (Clontech, Palo Alto, CA, USA). The sequences of primers used are shown in Supplementary Table 4. The PCR products were purified and cloned into the pMD18-T vector (Invitrogen). Six clones were randomly selected from each sample and sequenced [16].

### Cell culture

The human NSCLC cell lines, A549, NCI-H1395, NCI-H226, NCI-H460, and NCI-H520, were obtained from the American Type Culture Collection (ATCC, Manassas, VA, USA). All cell lines were routinely cultured under standard cell culture conditions (95% air and 5% CO_2_ at 37 °C). Cell line authentication was performed using the short tandem repeat (STR) DNA profiling method. Routine mycoplasma testing was performed by PCR.

### 5-Aza-2′-deoxycytidine treatment

NSCLC cells were treated with different concentrations (1, 2.5, and 5 μmol/L) of the DNA methyltransferase inhibitor, 5-aza-2′-deoxycytidine (5-Aza-dC, Sigma, St. Louis, MO, USA) or not treated with the 5-Aza-dC for three days. All media were changed daily.

### Real-time quantitative RT-PCR

The assay was performed as previously described [16]. The *ΔΔ*Ct method was used to determine the relative expression levels [11, 54]. The sequences of primers used are shown in Supplementary Table 4.

### Western blotting

Western blotting was performed according to a previously published study [12]. Anti-CD147 mAb was prepared in our lab [11]. Primary antibodies against Sp1 (sc-59), Krueppel-like factor 6 (KLF6, sc-365633), DNA methyltransferase 3A (DNMT3A, sc-373905), methyl-CpG-binding protein 2 (MeCP2, sc-137070), Tet1 (sc-293186), Tet2 (sc-398535), TDG (sc-376652), and β-actin (sc-8432) were purchased from Santa Cruz Biotechnology. Anti-SMAD2/3 mAb (ab63672) was purchased from Abcam (Abcam, Cambridge, UK) and horseradish peroxidase (HRP)-conjugated secondary antibodies were purchased from Thermo Fisher Scientific (31430 and 31460, Thermo Fisher Scientific, Waltham, MA, USA).

### Dot blot analysis

Genomic DNA was extracted and quantified, denatured in sodium hydroxide solution, and neutralized using ammonium acetate. The denatured DNA samples (5 μL) were manually spotted onto an Amersham Hybond-N+ membrane, and the membrane was then incubated with anti-5mC or 5hmC antibody overnight at 4 °C. The membrane was then probed with HRP-conjugated secondary antibody for 1 h at 25 °C. The signal was measured using an Amersham enhanced chemiluminescence system (Pierce, Rockford, USA).

### ChIP assay

ChIP, ChIP-ReChIP, and DNA immunoprecipitation assays were performed following standard protocols as previously described [28]. The primer sequences that were used are shown in Supplementary Table 4. AID, APE-1, DNA Ligase, and DNA pol β antibodies (sc-14680, sc-17774, sc-47703, and sc-48810, respectively) were purchased from Santa Cruz Biotechnology.

### Co-immunoprecipitation (co-IP)

The co-IP assay was conducted using a co-IP kit (Thermo Fisher Scientific, MA, USA). Briefly, cell lysates were prepared and immobilized with 10 μg of the affinity-purified antibody of each co-IP assay. Using the control agarose resin for preclearance, the cell lysates were then incubated under gentle mixing or rocking overnight at 4 °C. After elution of the columns, the eluted samples were examined by western blotting.

### Methylation-specific and unmethylation-specific PCR

Approximately 2 μg of the genomic DNA isolated from NSCLC cells was treated with sodium bisulfate and purified using a DNA clean-up kit (Promega, Madison, WI, USA). The purified DNA was used as the template for PCR. The primer specific for unmethylated DNA was *CD147*-U and the primer for methylated DNA was *CD147*-M [16]. All PCR primer sequences used in this study are listed in Supplementary Table 4.

### Construction of the targeted methylation vector

The dCas9-SunTag vector (60903) and scFv-GFP-DNMT3A vector (102278) were purchased from Addgene plasmid depository. The gRNA vectors for *CD147* were constructed by inserting the target sequences into a plasmid (57824, Addgene). The sequences of small guide RNAs (sg*CD147*) [55] sequences are listed in Supplementary Table 4. Cloning was performed by linearization and Gibson assembly-mediated incorporation of the gRNA insert fragment. We then generated lung cancer cells showing stable expression after lentiviral transduction of the constructs with pMD2.G and psPAX2 (12259 and 12260, Addgene), respectively.

### Cell proliferation assay

For cell-growth detection, 2 × 10^3^ cells were seeded into 96-well plates and cultured for 24, 48, 72, and 96 h. Subsequently, 20 μL of 5 mg/mL MTT solution (Sigma) was added to the wells, and the places were incubated for 4 h. Then, 150 μL dimethyl sulfoxide was added to the wells to dissolve the contents, and the optical density was measured at 490 nm.

### Colony-formation Assay

For clonogenicity analysis, 500 cells were seeded in six-well plates and cultured for 14 days. Then, the colonies were fixed with 4% paraformaldehyde for 30 min and stained using 0.05% crystal violet for 20 min. The numbers of colonies were counted.

### In vitro invasion and migration assays

An in vitro invasion assay was performed using 24-well MilliCell chambers (Millipore Corporation, Billerica, MA, USA) precoated with Matrigel (Falcon 354, BD Biosciences, USA) as described previously [11]. The protocol used for the migration assay was the same as that used for the invasion assay with the exception of the lack of matrigel coating and the cell permeation time, which was 12 h.

### Wound-healing assay

Cells were grown to nearly 90% confluence in six-well plates. A scratch wound was generated using a sterile plastic pipette tip and the cells were re-incubated for up to 24 h in serum-reduced medium containing 1% FBS. The wound areas were photographed at different time points and analyzed using ImageJ software (National Institutes of Health).

### Animal studies

Female BALB/c nude mice, aged 4–6 weeks, were provided by the Laboratory Animal Research Center of FMMU. All animal experiments were performed after the approval of the Animal Care and Use Committee of FMMU.

To study primary tumor growth, 2 × 10^6^ cells in 100 μL Matrigel stably expressing luciferase (Shanghai Sciencelight Biology Science & Technology Co., Ltd, Shanghai, China) were randomly orthotopically injected into the mammary fat pad of mice on Day 0. Anaesthetized mice were injected 150 mg/kg d-Luciferin Firefly potassium salt (Sciencelight) weekly via the intra-abdominal route, and data were acquired using Carestream MS FX Pro in vivo imaging system (Carestream Health, Cheektowaga, NY, USA). The region of interest data was analyzed using Carestream MI image analysis software. The bioluminescence signals were normalized to photons per second per millimeter squared (p/s/mm^2^). Subsequently, the mice were sacrificed, and the tumor tissues were collected and visually examined. The tumor volume was calculated using the formula length × width^2^/2 [56].

For metastasis studies, the airways of mice were preconditioned with the fibrosis-inducing agent, bleomycin, and 2 × 10^5^ NSCLC cells in 50 μL phosphate-buffered saline were injected into the airways of anesthetized mice [57]. At each week, the luminescence intensity was acquired. Bone damage was assessed using X-ray radiography. Osteolytic areas were identified as demarcated radiolucent lesions on radiographs and quantified using ImageJ software [56].

### Histological analysis

At the end of the experiment, the long bones of the forelimb and hind limb of nude mice were excised. Formalin-fixed, paraffin-embedded tumor tissue samples were serially sectioned into 4-μm-thick slices. After hematoxylin and eosin (H&E) staining, the sections were further examined by a pathologist to verify the tumor. Next, IHC analysis was performed using CD147 and Ki67 (sc-23900, Santa Cruz) antibodies.

### cfDNA isolation and analysis of CD147 methylation status of patients with NSCLC

Plasma cfDNA was isolated using the QIAamp Circulating Nucleic Acid kit (Qiagen) from 4 mL of peripheral blood samples from each patient collected in EDTA-containing anticoagulant tubes [49]. cfDNA was sonicated to generate 200–500 bp fragments. Approximately 200 ng of sonicated DNA was used to enrich the methylation fragments with a MethylCollector^TM^ Ultra Kit (Active Motif). The affinity of MBD2b for CpG-methylated DNA was increased by combining the His-tagged recombinant MBD2b and its binding partner MBD3L1. The sensitivity of this method is high, and the method can be used to enrich methylated DNA fragments with as few as five methylated CpG sites. Protein-DNA complexes were captured using nickel-coated magnetic beads. Specific quantitative PCR was performed with precipitated DNA using specific primers for the *CD147* promoter (Supplementary Table 4).

### Statistical analysis

Unless stated otherwise, all experiments were performed three to five times, and the data were expressed as the means ± SEMs. The statistical analyses were performed using SPSS 23.0 (IBM, Chicago, IL, USA) and GraphPad Prism 8 (GraphPad Software, Inc., San Diego, CA, USA). Student’s *t*-test was used to determine differences between groups, and one-way ANOVA was used for multiple groups. Two-way ANOVA and Bonferroni tests were used to analyze the in vitro and in vivo proliferation results. The overall survival of mice injected with targeted methylation of *CD147* cells was calculated by Kaplan–Meier analysis. The Spearman rank correlation coefficient was used as a statistical measure of association. All statistical tests were two-sided, and *P* values < 0.05 were considered to indicate statistical significance.

## Supplementary information

Supplementary material

Supplementary Figure 1

Supplementary Figure 2

Supplementary Figure 3

Supplementary Figure 4

Supplementary Figure 5

Supplementary Figure 6

Supplementary Table 1

Supplementary Table 2

Supplementary Table 3

Supplementary Table 4
