# Supplementary material for: Active demethylation upregulates CD147 expression promoting non-small cell lung cancer invasion and metastasis
Source: Oncogene. 2022 Feb 7;41(12):1780–94. doi: 10.1038/s41388-022-02213-0 (PMC8933279; doi:10.1038/s41388-022-02213-0)
Supplement: Supplementary file 1 — Supplementary material [file 41388_2022_2213_MOESM1_ESM.docx]

**Supplementary methods**

**Immunohistochemical analysis**

Immunohistochemistry was performed using a CD147 diagnostic kit according to the manufacturer’s manual (Jiangsu Pacific Meniuoke Biopharmaceutical Company, Changzhou, China). The expression level was independently evaluated by two experienced pathologists blind to the study design. Expression of CD147 was evaluated according to the formula: overall score = percentage score × intensity score. The ratio of positive cells per specimen was evaluated quantitatively and scored as 0 for staining of ≤ 10%, 1 for staining of 11 to 25%, 2 for staining of 26 to 50%, 3 for staining of 51 to 75%, and 4 for staining > 75% of the cells examined. Intensity was graded as follows: 0, no signal; 1, weak; and 2, strong staining. A total score of 0 to 8 was calculated and graded as negative (-; score: 0-1), positive: weak (+; score: 2-4), and strong (++; score: 6-8) [1-3].

**Supplementary Figures legends:**

**Supplementary Figure 1:** The results of MeDIP and hMeDIP Analysis. **(A)** Genomic analysis of 5mC and 5hmC location in four paired adjacent normal tissues and NSCLC samples. Pie chart indicating the genomic distribution of 5mC and 5hmC location in four paired adjacent normal tissues and NSCLC samples. **(B)** Overlap between 5mC and 5hmC location. Venn diagram indicating the number of genes annotated for 5mC and 5hmC. Shown in the square are selected genes.

**Supplementary Figure 2:** The correlations between CD147 expression and the content of 5mC\5hmC in the *CD147* promoter.

**Supplementary Figure 3:** The CD147 expression and methylation levels in tumor cells. **(A)** The CD147 expression were detected by western blot. **(B)** Methylation profiles of CpG dinucleotides in the *CD147* promoter in tumor cells detected using bisulfite genomic sequencing (BGS).

**Supplementary Figure 4:** The CD147 protein expression in NSCLC cells detected by Western blot.

**Supplementary Figure 5:** Knockdown of DNMT3A or Tet1 using siRNA. Western blot analysis was performed to detect the protein expression with the indicated siRNA in A549 and NCI-H460 cells. Right panel, the *CD147* promoter methylation status after the knockdown by BGS.

**Supplementary Figure 6:** The correlation between *CD147* methylation in cfDNA and CD147 expression in primary lung tissues. **(A)** Representative immunohistochemistry staining of CD147 in primary NSCLC tissues. **(B)** Analysis of the correlation between *CD147* methylation levels in cfDNA and CD147 expression in NSCLC tissues via Spearman’s rank correlation coefficient.

**Reference**

1. Fei, F., et al., *CD147-CD98hc complex contributes to poor prognosis of non-small cell lung cancer patients through promoting cell proliferation via the PI3K/Akt signaling pathway.* Ann Surg Oncol, 2014. **21**(13): p. 4359-68.

2. Kong, L.M., et al., *Transcription factor Sp1 regulates expression of cancer-associated molecule CD147 in human lung cancer.* Cancer Sci, 2010. **101**(6): p. 1463-70.

3. Kong, L.M., et al., *A regulatory loop involving miR-22, Sp1, and c-Myc modulates CD147 expression in breast cancer invasion and metastasis.* Cancer Res, 2014. **74**(14): p. 3764-78.
