# Supplementary figures and images for: Active demethylation upregulates CD147 expression promoting non-small cell lung cancer invasion and metastasis
Source: Oncogene. 2022 Feb 7;41(12):1780–94. doi: 10.1038/s41388-022-02213-0 (PMC8933279; doi:10.1038/s41388-022-02213-0)

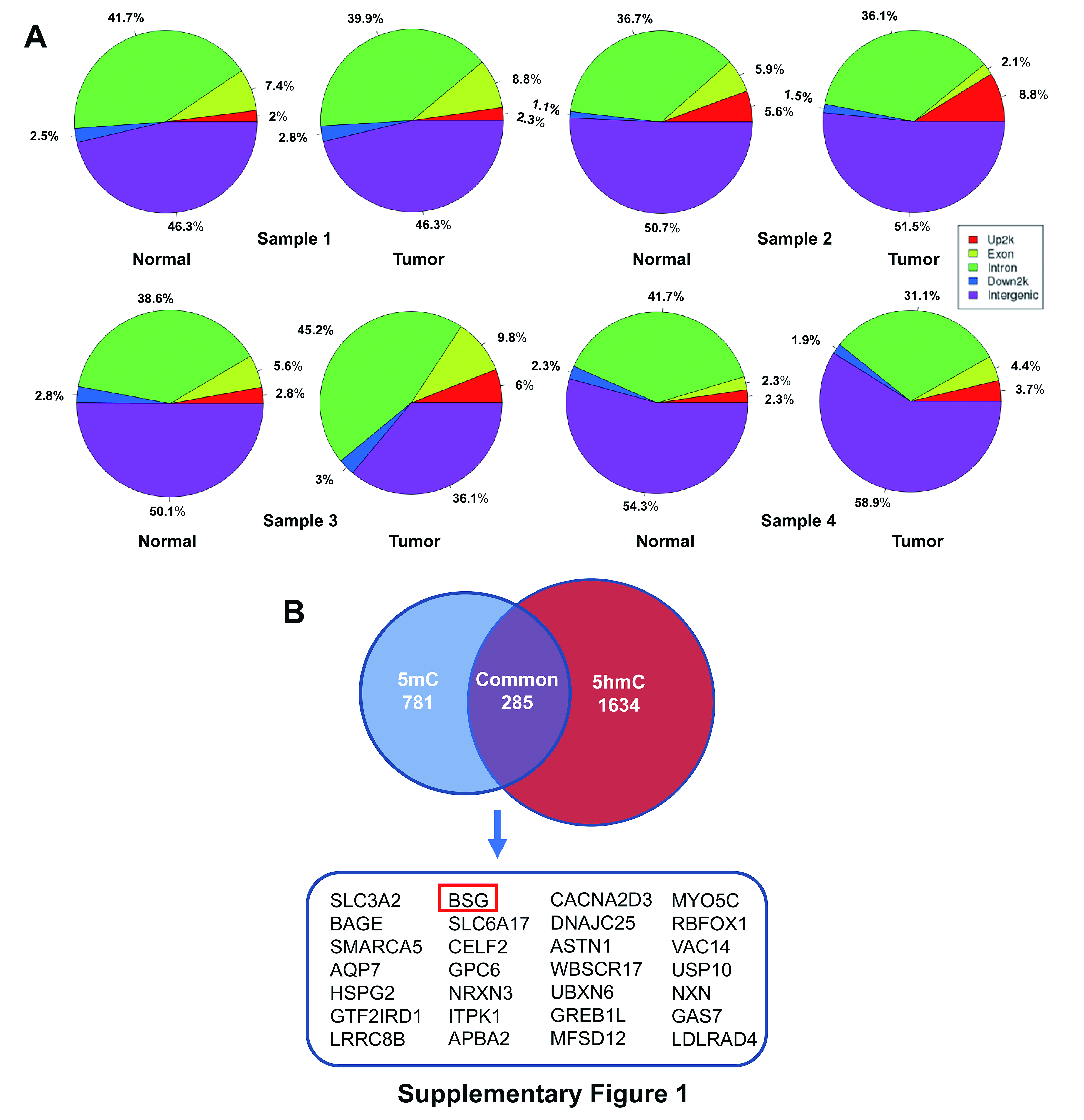

Supplement: Supplementary file 2 — Supplementary Figure 1 [file 41388_2022_2213_MOESM2_ESM.tif]

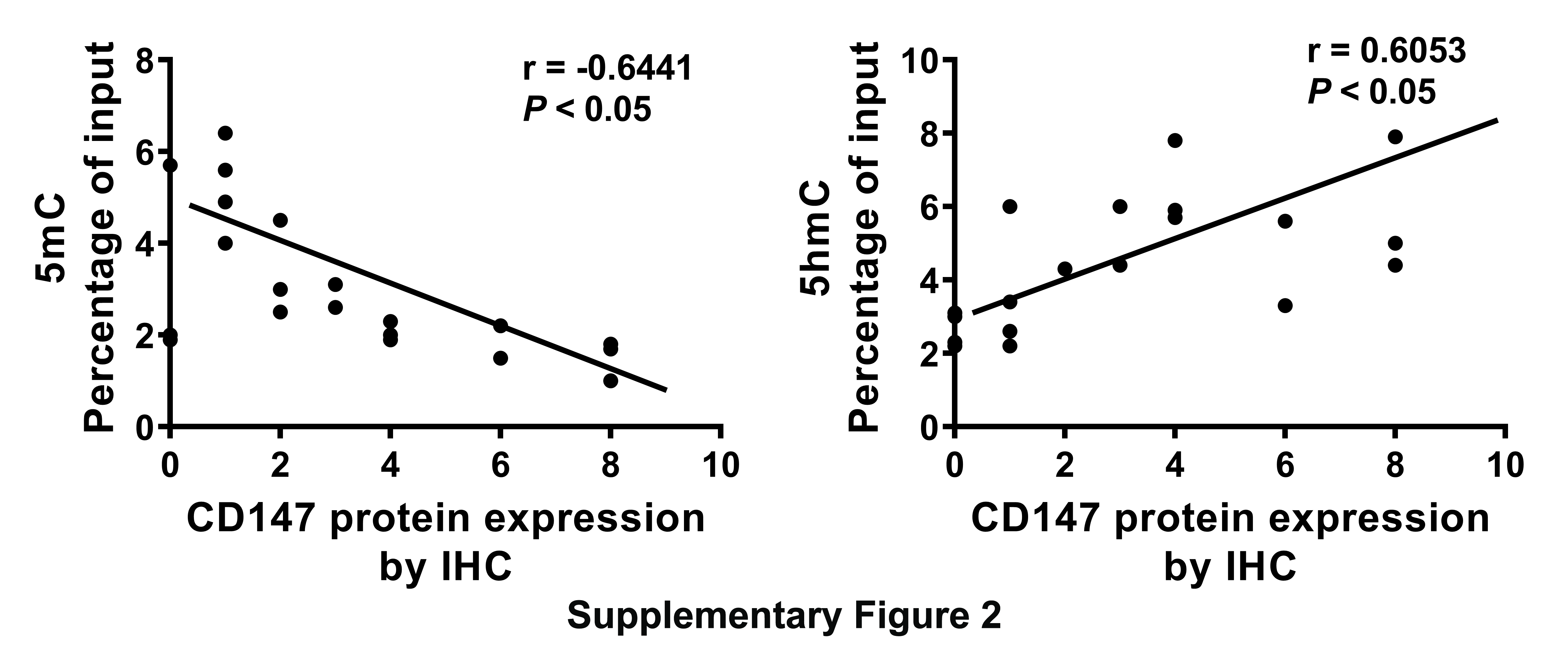

Supplement: Supplementary file 3 — Supplementary Figure 2 [file 41388_2022_2213_MOESM3_ESM.tif]

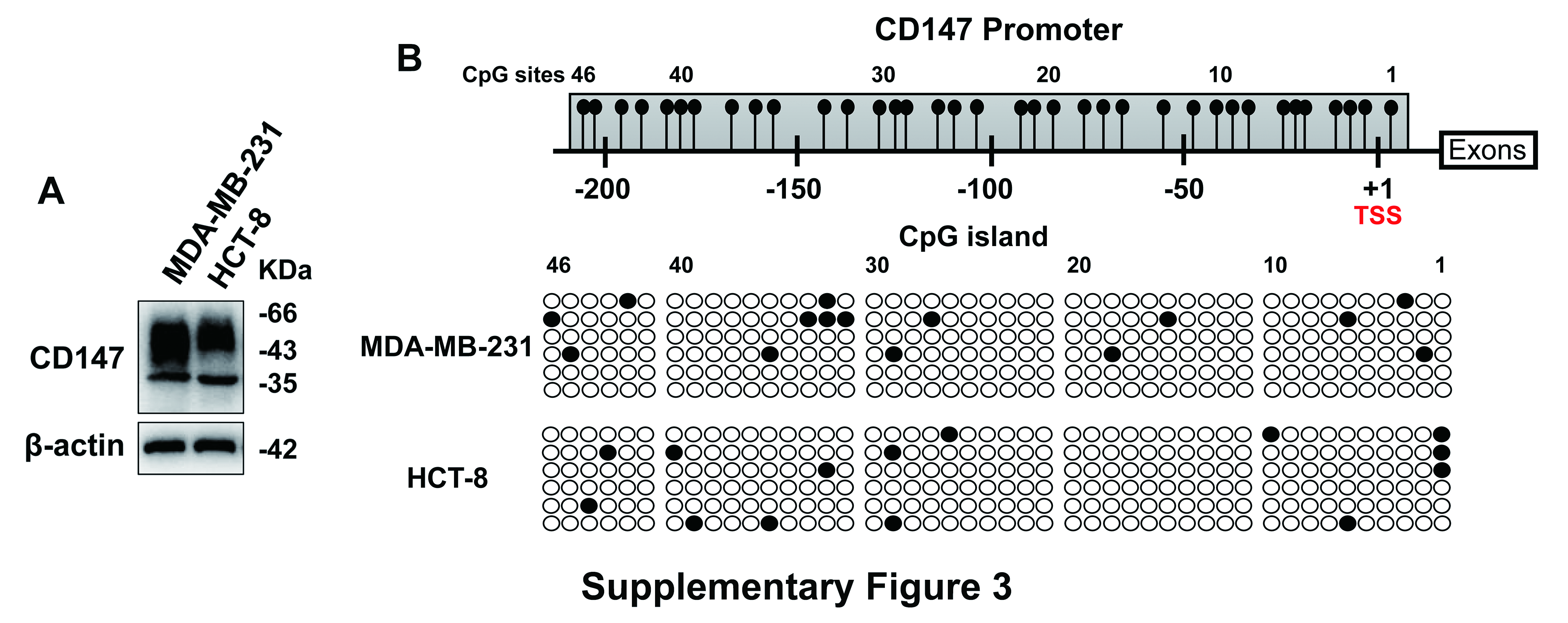

Supplement: Supplementary file 4 — Supplementary Figure 3 [file 41388_2022_2213_MOESM4_ESM.tif]

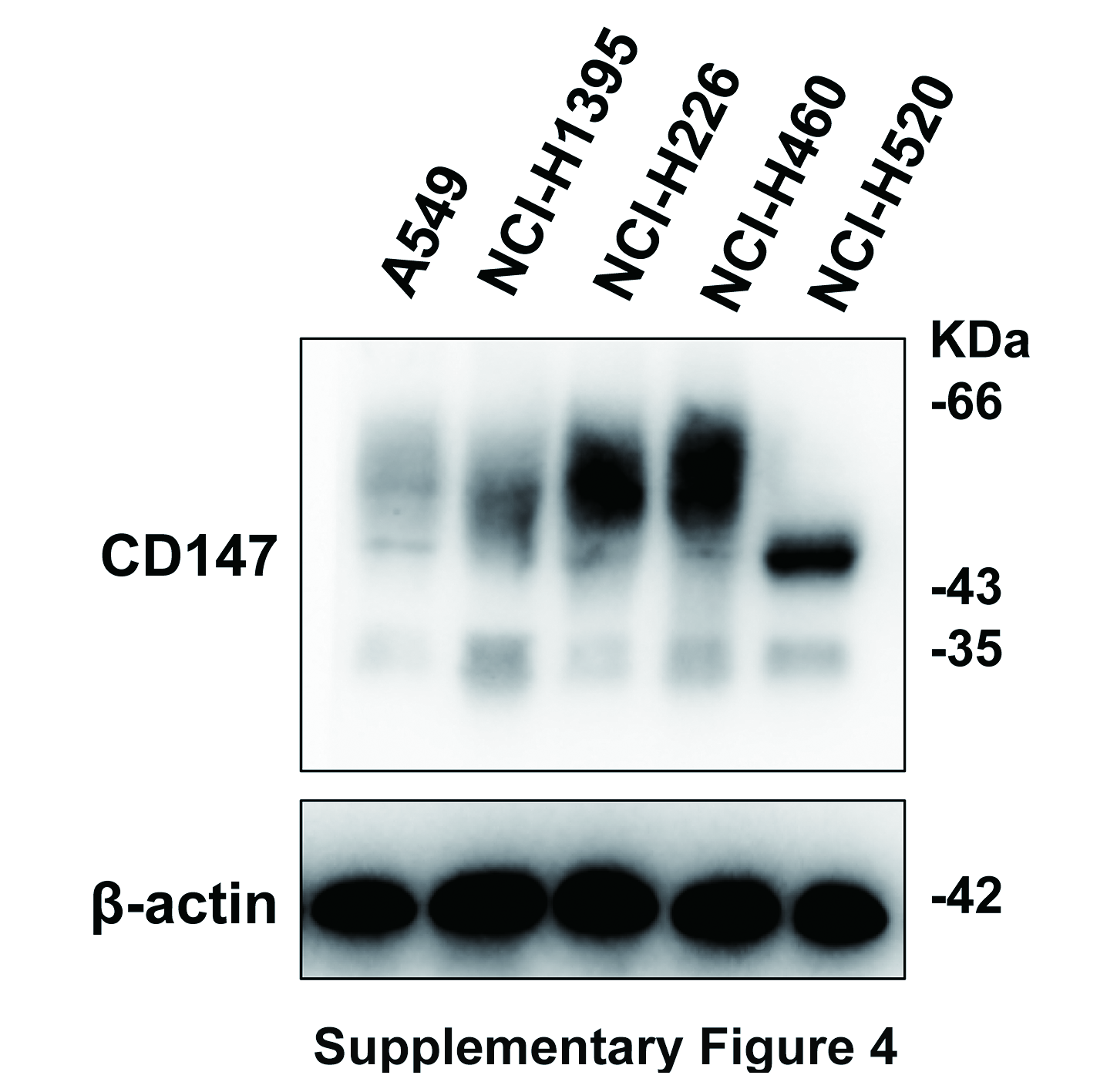

Supplement: Supplementary file 5 — Supplementary Figure 4 [file 41388_2022_2213_MOESM5_ESM.tif]

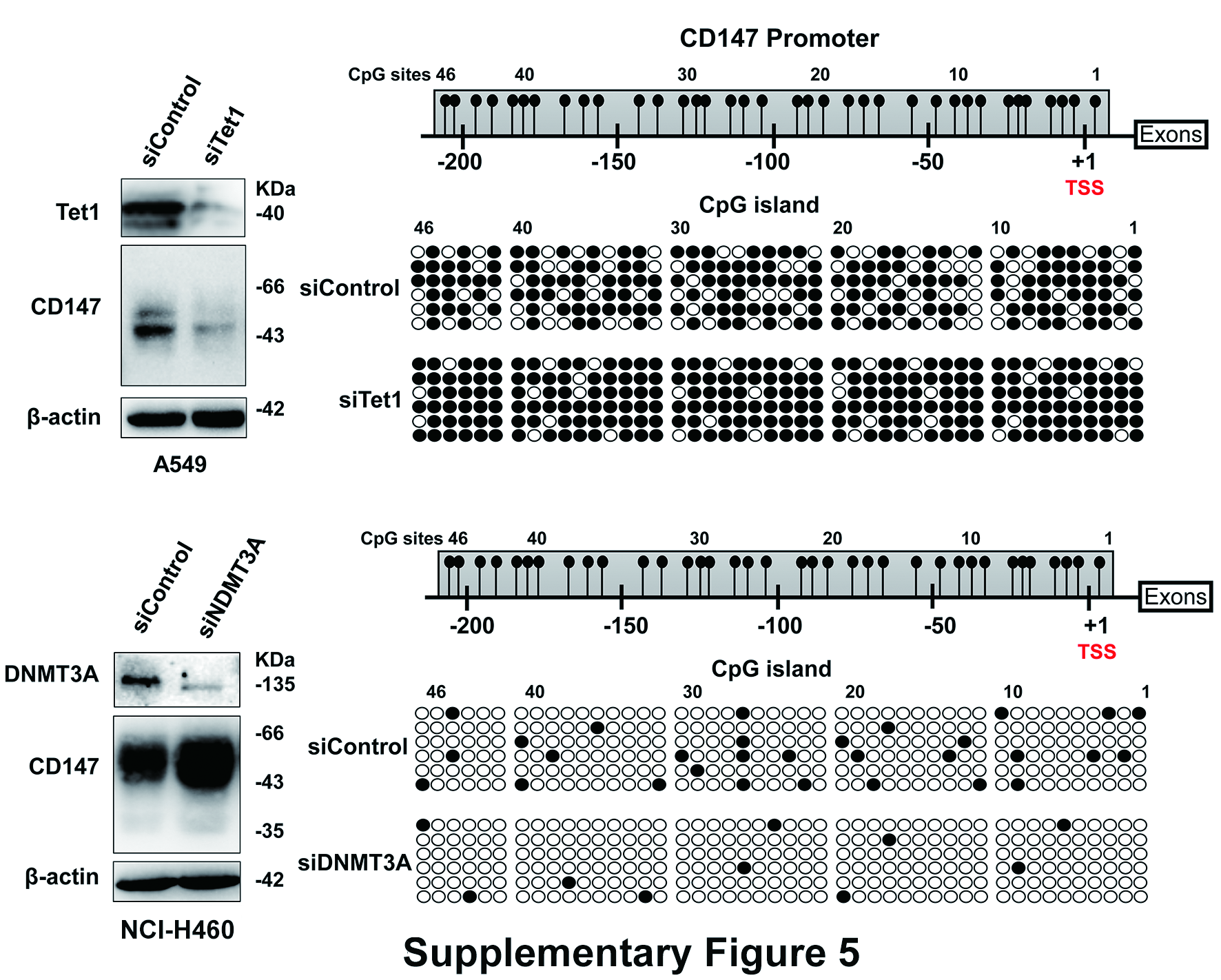

Supplement: Supplementary file 6 — Supplementary Figure 5 [file 41388_2022_2213_MOESM6_ESM.tif]

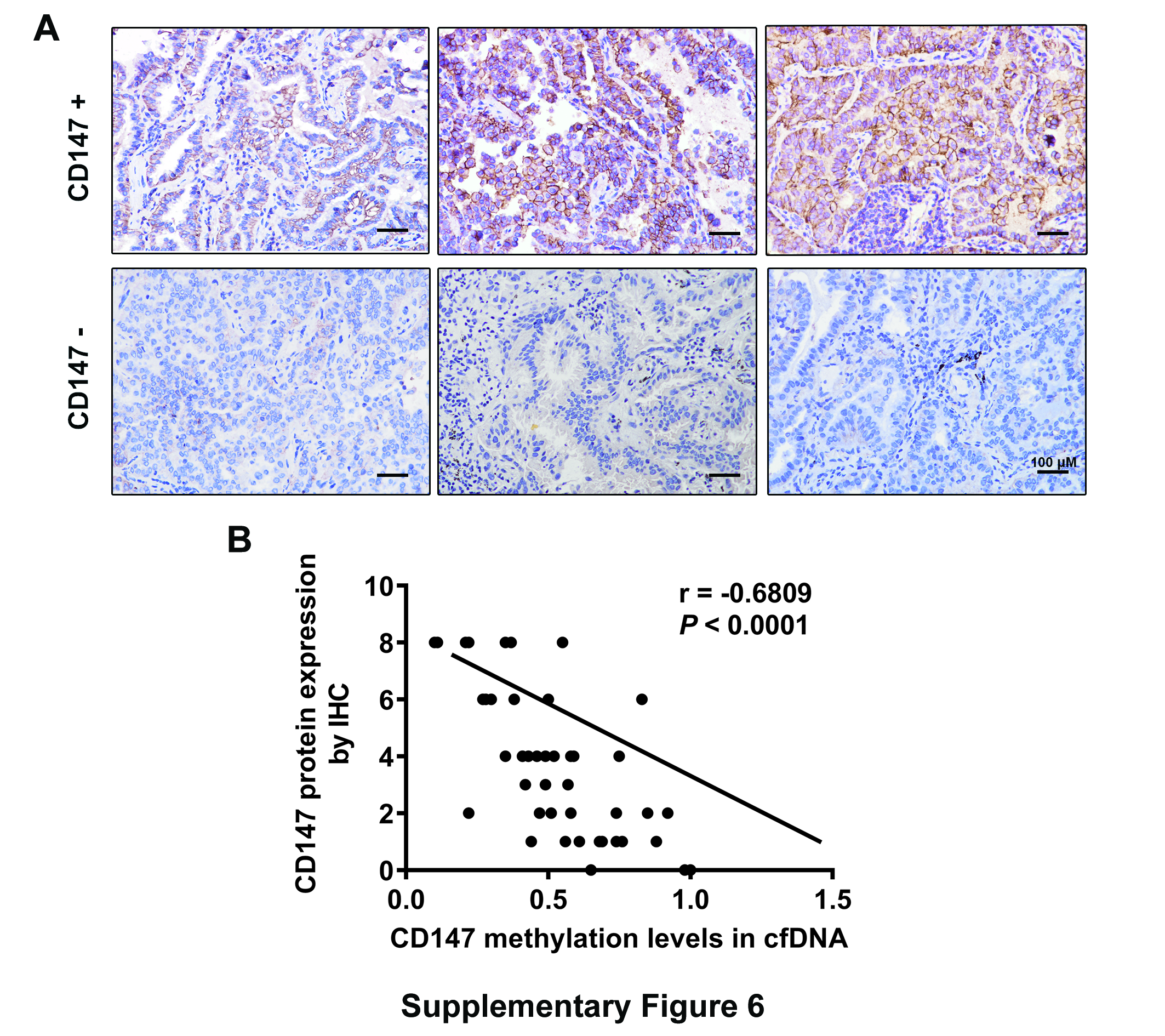

Supplement: Supplementary file 7 — Supplementary Figure 6 [file 41388_2022_2213_MOESM7_ESM.tif]
